# ﻿Four new species of the genus *Xynobius* Foerster (Hymenoptera, Braconidae, Opiinae) from South Korea

**DOI:** 10.3897/zookeys.1193.115831

**Published:** 2024-03-07

**Authors:** Yunjong Han, Cornelis van Achterberg, Hyojoong Kim

**Affiliations:** 1 Animal Systematics Laboratory, Department of Biological Science, Kunsan National University, Gunsan, 54150, Republic of Korea Kunsan National University Gunsan Republic of Korea; 2 Naturalis Biodiversity Center, P.O. 9517, 2300 RA Leiden, Netherlands Naturalis Biodiversity Centre Leiden Netherlands

**Keywords:** Description, identification, key, parasitoid, Republic of Korea

## Abstract

Four new species, *Xynobiusazonius***sp. nov.**, *X.brevifemora***sp. nov.**, *X.duoferus***sp. nov.**, and *X.stipitoides***sp. nov.**, are described and illustrated, and one species *X.geniculatus* (Thomson, 1895) is newly reported from South Korea. *Xynobiusgeniculatus* (Thomson, 1895) is redescribed and illustrated, and a new combination, Xynobius (Stigmatopoea) cubitalis (Fischer, 1959), **comb. nov.** is suggested. An identification key to the *Xynobius* species known from South Korea is provided.

## ﻿Introduction

The large and cosmopolitan subfamily Opiinae Blanchard, 1845 consists of derived koinobiont cyclostome wasps, with approximately 2,100 described valid species to date (Yu et al. 2016). Members of the Opiinae are koinobiont endoparasitoids of dipterous larvae, of which some are agricultural pests such as leaf miners and fruit feeders. Therefore, their opiine parasitoids might be valuable for biological control (Wharton 1997; Ovruski et al. 2000; Delrio et al. 2005; Wahyuni et al. 2017). The taxonomy of the Opiinae is still subject to much discussion and fluctuation because of their intermediate characters. The number, validity, and systematic placement of several genera is not yet finalised, and debates exist regarding the classification of certain genera, such as *Opius* Wesmael, 1835 and *Eurytenes* Foerster, 1863 (e.g., Wharton 1987, 1988, 1997; Wharton and Norrbom 2013) and some new genera (e.g., van Achterberg 2023). Papp (1981a, 1989, 1992) reported *Xynobiustenuicornis* (Thomson, 1895), *X.japanus* (Fischer, 1963), *X.caelatus* (Haliday, 1837), and *X.comatus* (Wesmael, 1835) from North Korea, and the national species list of Korea (National Institute of Biological Resources (NIBR), 2019) lists five species, including *X.rudis* Wesmael, 1835. However, *X.rudis* belongs to a different genus, viz., *Neopius* Gahan, 1917 according to Sheng et al. (2019). In addition, the reported species *Apodesmiasapporanus* (Fischer, 1963) belongs to the genus *Xynobius* (Han et al. 2023) and should be included in this review.

We treat *Xynobius* Foerster, 1863 as a valid genus separate from *Opius* following Li et al. (2013) and van Achterberg (2023). We report five *Xynobius* species, four new to science and one newly recorded in South Korea. An identification key to the Korean *Xynobius* is provided with descriptions and illustrations of the new species.

## ﻿Materials and methods

Specimens of *Xynobiusazonius* sp. nov., *X.duoferus* sp. nov., and *X.stipitoides* sp. nov. were collected by using a net to sweep the herbal vegetation, while those of *X.brevifemora* sp. nov. and *X.geniculatus* were collected in a Malaise trap. All specimens were preserved in 70% alcohol. For identification of the subfamily Opiinae, see van Achterberg (1990, 1993, and 1997); for references to the classification of the Opiinae, see Yu et al. (2016).

Morphological terminology follows van Achterberg (1988, 1993), including the abbreviations for the wing venation. Measurements were taken as indicated by van Achterberg (1988); for the length and the width of a body part the maximum length and width is taken, unless otherwise indicated. The length of the mesosoma is measured from the anterior border of the mesoscutum to the apex of the propodeum and of the first tergite from the posterior border of the adductor to the medio-posterior margin of the tergite.

Observations, photographic images, and descriptions were made either with a LEICA DMC2900 digital camera or with a LEICA M205 C microscope (Leica Geosystems AG). The photos were stacked with Helicon Focus v. 7 software (Helicon Soft, Kharkiv, Ukraine) After stacking, illustrations were created using Adobe Photoshop CS5.1.

The holotype of *Xynobiusduoferus* sp. nov. is deposited in the National Institute of Biological Resources (**NIBR**) at Incheon and the specimen of *X.geniculatus* (Thomson, 1895) is deposited in the Korea National Arboretum (**KNA**) at Pocheon. The remaining holotypes are deposited in the Kunsan National University (**KSNU**) at Gunsan.

## ﻿Systematics

### ﻿Genus Xynobius

Taxon classificationAnimaliaHymenopteraBraconidae

Foerster, 1863

0C032E95-3A93-5168-AEE3-4785094E5A8C

Xynobius Foerster, 1863: 235. Type species (by original designation): Xynobiuspallipes Foerster, 1863 (= Opiuscaelatus Haliday, 1837).
Aclisis Foerster, 1863: 267. Type species (by original designation): Aclisisisomera Foerster, 1863 (= Opiuscaelatus Haliday, 1837). Synonymised by Fischer (1972).
Holconotus Foerster, 1863: 259 (not Schmidt-Göbel, 1846). Type species (by original designation): Opiuscomatus Wesmael, 1835). Synonymised by van Achterberg (2004).
Aulonotus Ashmead, 1900: 368 (new name for Holconotus Foerster). Type species (by original designation): Opiuscomatus Wesmael, 1835). Synonymised by Tobias and Jakimavičius (1986).
Eristernaulax Viereck, 1913: 362. Type species (by original designation): Eristernaulaxleucotaenia Viereck, 1913). Synonymised by van Achterberg (2004).
Stigmatopoea Fischer, 1984: 610, 611 (as subgenus of Opius Wesmael), 1998: 25 (key to species); Wharton, 1988: 356; 2006: 338 (as subgenus of Eurytenes Foerster, 1863; possible paraphyly in Xynobius). Type species (by original designation): Opiusmacrocerus Thomson, 1895. Synonymised by van Achterberg (2004).
Xynobiotenes Fischer, 1998: 23 (as subgenus of Eurytenes Foerster, 1863). Type species (by original designation): Opiusscutellatus Fischer, 1962. Synonymised by Li et al. (2013).

#### Diagnosis.

Hypoclypeal depression distinct and ventral margin of clypeus above upper level of mandibular condyles (Figs 6, 20, 34, 48, 58, 70); mandible without acute basal lamella; notauli complete (Fig. 32) or largely absent except a pair of short anterior impressions (Figs 4, 16, 57); medio-posterior depression variable, often present (Figs 4, 32, 45, 57, 67); precoxal sulcus distinct and no sternaulus; vein r of fore wing more or less angled with vein 3-SR and distinctly shorter than vein 2-SR (Figs 2, 14, 30, 43, 55, 65); pterostigma either narrowed apically or parallel-sided to slightly widened apically (subgenus Stigmatopoea: Figs 55, 65); dorsope distinct (Figs 5, 9, 22, 25, 26, 37, 45, 46, 50, 51, 57, 58, 68, 69).

#### Distribution.

Cosmopolitan.

#### Biology.

Koinobionts endoparasitoids of mining dipterous larvae (species of the genus *Agromyza* Fallen, 1810; Agromyzidae), or of fruit-infesting larvae (species of the genera *Euliea* Walker, 1835, and *Trypeta* Meigen, 1803; Tephritidae).

### ﻿Key to Korean species of the genus *Xynobius* Foerster

The number of included species for Korea is based on the list by Yu et al. (2016), the Korean species list (NIBR 2019), and this study; only *X.cubitalis* (Fischer, 1959) is included as a new combination.

**Table:** 

1	Pterostigma subparallel-sided and more or less widened apically (Figs 43, 65); subgenus Stigmatopoea Fischer, 1986	**2**
–	Pterostigma elliptical or triangular, narrowed apically (Figs 2, 14, 30, 55); subgenus Xynobius Foerster, 1863	**3**
2	Propodeum finely and weakly rugose; medio-posterior depression of mesoscutum round; face weakly punctate and rugose; length of hind femur 6–7× its width; third and following metasomal tergites without pale bands	**X. (S.) cubitalis (Fischer, 1959), comb. nov.**
–	Propodeum mainly coarsely rugose; medio-posterior depression of mesoscutum sublinear; face densely punctate; length of hind femur 4.7× its width; third and following metasomal tergites with pale transverse bands [notauli present up to middle of mesoscutum and narrowly crenulate; vein SR1 of fore wing 2.7× longer than vein 3-SR; first tergite subparallel-sided and nearly twice longer than its apical width]	**X. (S.) stipitoides Han & van Achterberg, sp. nov.**
3	Vein m-cu of fore wing antefurcal or interstitial [rarely in *X.sapporanus*; see couplet 10]	**4**
–	Vein m-cu of fore wing postfurcal	**7**
4	Scutellum smooth; face conspicuously setose (Figs 20, 21, 27)	**5**
–	Scutellum entirely sculptured; face inconspicuously setose	**6**
5	Notauli absent on mesoscutal disc; pronope absent; antenna of ♀ with 40 segments; middle lobe of mesoscutum largely glabrous; face punctate; medio-posterior depression of mesoscutum absent [occiput smooth with setae; all femora robust; wing rather infuscated; mesosoma except metapleuron and propodeum orange-brown]	**X. (X.) brevifemora Han & van Achterberg, sp. nov.**
–	Notauli at least present on anterior half of mesoscutal disc; pronope present; antenna of ♀ with 22–24 segments; middle lobe of mesoscutum evenly setose; face smooth; medio-posterior depression of mesoscutum present, round [propodeum reticulate-rugose]	**X. (X.) comatus (Wesmael, 1835)**
6	Scutellum densely rugose; occiput punctate; antenna with 41–50 segments [notauli complete and narrowly crenulate; middle lobe of mesoscutum faintly punctate; medio-posterior depression of mesoscutum round and surrounding area rugulose; pronotal side extensively rugose expect dorsally; ventral margin of clypeus concave]	**X. (X.) caelatus (Haliday, 1837)**
–	Scutellum coarsely punctate; occiput smooth or with some fine punctures; antenna with 50–54 segments	**X. (X.) japanus (Fischer, 1963)**
7	Antenna of ♀ with white subapical band; second metasomal tergite striate-rugose medially [first tergite with straight longitudinal striae; notauli complete and narrowly crenulate; mesoscutum largely smooth and sparsely setose medially; antenna with two dark apical segments]	**X. (X.) duoferus Han & van Achterberg, sp. nov.**
–	Antenna of ♀ without white subapical band and dark brown to brownish subapically; second tergite smooth or finely striate	**8**
8	First metasomal tergite 1.7–2.2× longer than its apical width; second tergite more or less finely striate; antenna with 28–31 segments [frons, vertex and entire occiput blackish brown; propodeum smooth or only carinate]	**X. (X.) tenuicornis (Thomson, 1895)**
–	First tergite 1.3–1.5× longer than its apical width; second tergite smooth; antenna with 35–44 segments	**9**
9	Precoxal sulcus smooth [malar sulcus distinct and deep; antenna with 38–44 segments; area below pterostigma with brownish patch, rarely obsolescent; second submarginal cell of fore wing long; second metasomal tergite bicoloured (dark brown and with a pale yellowish patch medially); hind tarsus pale yellowish or ivory; apex of hind femur dark brown]	**X. (X.) geniculatus (Thomson, 1895)**
–	Precoxal sulcus sculptured	**10**
10	Antenna with 39–42 segments; area below pterostigma with a large Y-shaped dark brownish patch; notauli largely impressed on mesoscutal disc [vertex and mesoscutum conspicuously setose; vein m-cu of fore wing variable, usually postfurcal; medio-posterior depression of mesoscutum elongated; propodeum and first tergite coarsely rugose]	**X. (X.) sapporanus (Fischer, 1963)**
–	Antenna with 35 segments; area below pterostigma subhyaline; notauli absent on mesoscutal disc [malar sulcus absent; 5^th^–7^th^ tergites yellow posteriorly without dark brown band; vein r and 2-SR of fore wing ~ 0.7 and 2.5× as long as vein m-cu, respectively]	**X. (X.) azonius Han & van Achterberg, sp. nov.**

### ﻿ Xynobius azonius

Taxon classificationAnimaliaHymenopteraBraconidae

Han & van Achterberg sp. nov.

F7C9A319-526B-5C35-AA66-B1E356C4D16A

https://zoobank.org/C76FAE32-149D-4742-91E2-F2D577AE2215

Figs 1 , 2–12

#### Type material.

***Holotype*.** ♀ (KSNU), “South Korea: Amnam, Seo-gu, Busan, 35°04'48.6"N, 129°00'59.2"E, 14.v.2020, SW [= collected by sweeping], Hyojoong Kim leg., KSNU”.

**Figure 1.**
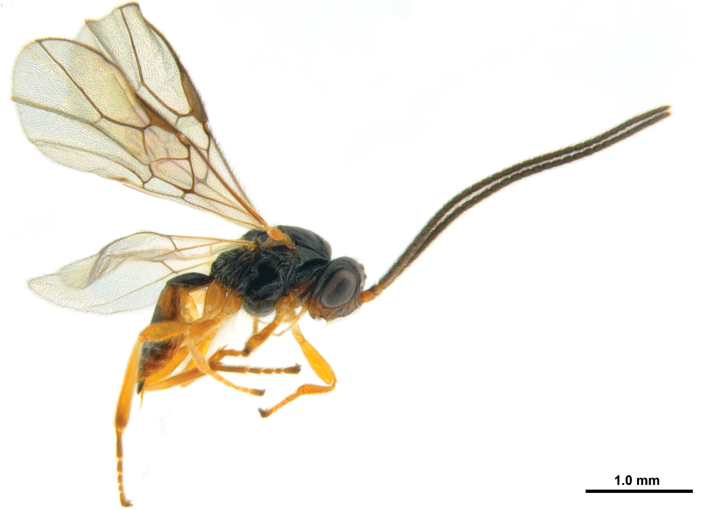
*Xynobiusazonius* Han & van Achterberg sp. nov., holotype, ♀, habitus, lateral.

#### Diagnosis.

**Figures 2–12.**
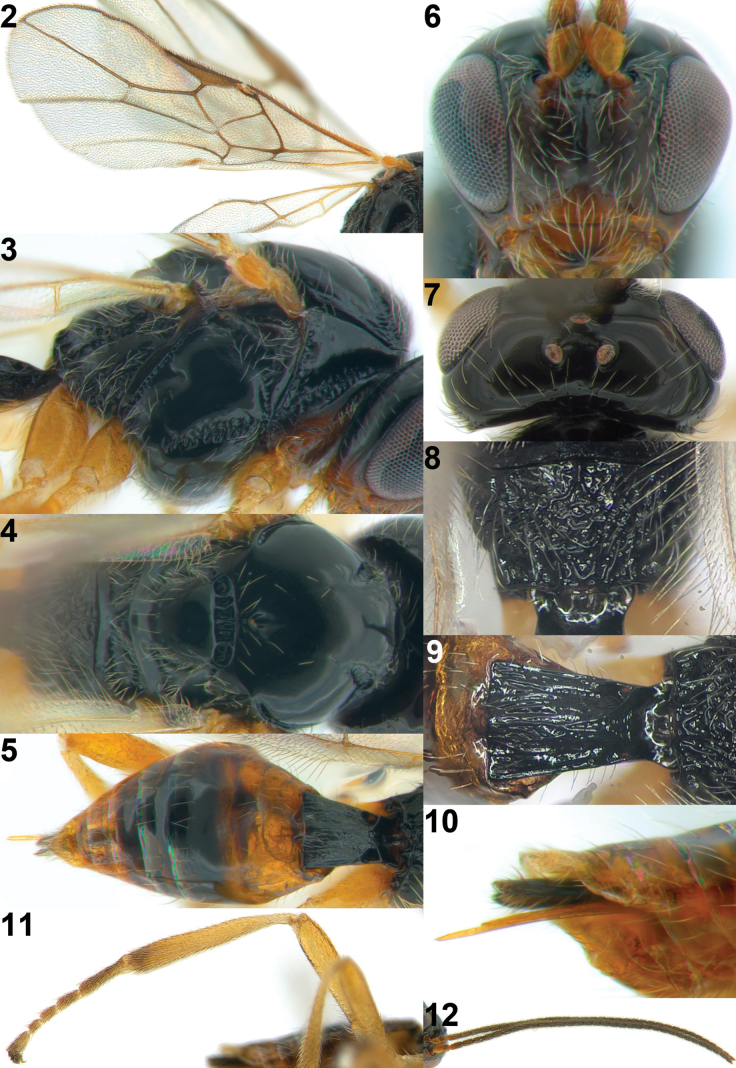
*Xynobiusazonius* Han & van Achterberg sp. nov., holotype, ♀ **2** wings **3** mesosoma, lateral view **4** mesosoma, dorsal view **5** metasoma, dorsal view **6** head, anterior view **7** head, dorsal view **8** propodeum, dorsal view **9** 1^st^ metasomal tergite, dorsal view **10** ovipositor and sheath, latero-ventral view **11** hind leg **12** antenna.

Antennal segments of ♀ ~ 35 and subapical segments dark brown; frons laterally and temple in dorsal view black; eye 1.6× longer than temple in dorsal view (Fig. 7); precoxal sulcus coarsely crenulate (Fig. 3); notauli absent on mesoscutal disc; pterostigma elliptical (Fig. 2); veins r and 2-SR of fore wing ~ 0.7 and 2.5× as long as vein m-cu, respectively; fore wing subhyaline; first metasomal tergite ~ 1.4× longer than its apical width (Fig. 5); second tergite smooth; fifth-seventh metasomal tergites yellow posteriorly, without apical dark brown band; ovipositor sheath short and comparatively robust (Fig. 10).

#### Description.

Female; length of body nearly 2.9 mm, of fore wing 3.0 mm.

***Head*.** Antenna with 35 segments and 1.1× as long as body (Fig. 12); third segment of antenna 2× longer than wide, as long as fourth segment of antenna; eye 1.6× longer than temple in dorsal view (Fig. 7); stemmaticum shiny and smooth; vertex shiny, smooth and moderately setose posteriorly; frons with depression medially and remainder shiny and smooth; face shallowly punctate and densely setose (Fig. 6); median keel present up to between antennal sockets; clypeus 2.3× wider than its maximum height; clypeus smooth and densely setose, protruding in lateral view; hypoclypeal depression present; malar sulcus absent; occipital carina absent medio-dorsally; mandible twisted, triangular in lateral view and gradually widened basally.

***Mesosoma*.** Mesosoma 1.4× longer than its height (Fig. 3); pronope elliptical and deep (Figs 4, 7); propleuron largely smooth and propleuron flange protruding posteriorly (Fig. 3); mesopleuron largely shiny and smooth, but precoxal sulcus crenulate, wide and reaching epicnemial area; epicnemial area distinctly crenulate; pronotal side largely smooth with crenulate groove anteriorly and posteriorly; mesopleural sulcus crenulate; anterior groove of metapleuron crenulate; metapleuron coarsely rugose and densely setose; notauli absent on disc of mesoscutum, except deep and crenulate impressions anteriorly (Fig. 4); mesoscutum shiny, smooth and sparsely setose along imaginary notaulic courses and around medio-posterior depression; scutellum shiny, smooth and rather convex; medio-posterior depression of mesoscutum round; scutellar sulcus crenulate, medium-sized; propodeum sparsely setose with short medio-longitudinal carina anteriorly, transverse carinae, areola, and remainder area shiny and smooth (Figs 4, 8, 9); inside of areola of propodeum reticulate-rugose.

***Wings*.** Fore wing (Fig. 2): pterostigma elliptical and narrowed apically; vein 1-M curved; vein 1-SR+M slightly sinuate; vein 3-SR angled with vein r, converged with vein 2-M and 1.6× longer than vein 2-SR; vein 2-SR straight and oblique; vein SR1 straight; r: 3-SR: SR1 = 5: 21: 41; vein m-cu postfurcal; vein CU1b medium-sized; first subdiscal cell closed. Hind wing: vein m-cu pigmented and curved basally; vein 1r-m 0.5× as long as vein 1-M; vein 2-M only pigmented.

***Legs*.** Hind femur 3.9× longer than its maximum width (Fig. 11).

***Metasoma*.** First metasomal tergite 1.4× longer than its apical width (Fig. 9); first tergite slightly widened apically, dorsal carinae converging medially and its surface with longitudinal striate; dorsope distinctly developed (Figs 5, 9); second metasomal suture superficially indicated dorsally (Fig. 5); second tergite shiny and smooth with a pair of oblique depressions anteriorly; following tergites shiny, smooth, with subposterior row of setae; setose part of ovipositor sheath 0.3× as long as first metasomal tergite (Figs 1, 10).

***Colour*.** Body, black; scape of antenna, clypeus, mandible, propleuron, second, sixth, and seventh tergites brown; flagellar segments of antenna, hind tibia, and tarsus dark brown; remainder of legs and palpi pale yellowish; pterostigma and vein of wings greyish brown; wings subhyaline.

#### Distribution.

South Korea.

#### Biology.

Unknown.

#### Etymology.

The new species is named for not having the isolated dark brown bands on the fourth–seventh tergites as in the similar *X.polyzonius* (Wesmael, 1835); “a” is Greek for not and “zone” is Greek for girdle or band.

#### Remarks.

This species runs to the genus *Xynobius* Foerster because of the dorsope at the base of the first tergite, vein 3-SR of the fore wing distinctly longer than vein 2-SR, the mandible more or less twisted medially, symmetrical basally and its second tooth hardly or not visible in lateral view, the hypoclypeal depression distinctly developed and the propleuron without oblique carina (van Achterberg 2023). However, it does not run well in the key by Tobias (1998) by having the notauli reduced (absent on mesoscutal disc and only a pair of crenulated impressions anteriorly), the mesoscutum smooth and sparsely setose, the medio-longitudinal carina and areola on the propodeum (with inner area of areola coarsely rugose), the first metasomal tergite comparatively stout (1.3× longer than its apical width) and the smooth and yellowish brown second tergite. Actually, the new species is similar to *X.polyzonius* (Wesmael, 1835) from which is differs by having ~ 35 antennal segments (♀: 24–31 in *X.polyzonius*), frons laterally and temples in dorsal view black (yellowish brown); veins r and 2-SR of fore wing ~ 0.7× and 2.5× as long as vein m-cu, respectively (0.5× and 1.6×, respectively) and fifth–seventh metasomal tergites without dark brown apical band (isolated bands present).

### ﻿ Xynobius brevifemora

Taxon classificationAnimaliaHymenopteraBraconidae

Han & van Achterberg sp. nov.

4E646E7A-82FF-59A8-A602-82A48702D5DF

https://zoobank.org/4679E4C0-BF7C-437B-80C0-70530160D032

Figs 13 , 14–24 , 25–28

#### Type material.

***Holotype*.** ♀ (KSNU), “South Korea: 290-2 Singwan-dong, Gunsan, Jeonbuk prov., 35°56'34"N, 126°40'45"E, 14.–30.v.2016, MT [= Malaise trap], Hyojoong Kim leg., KSNU”.

#### Diagnosis.

**Figure 13.**
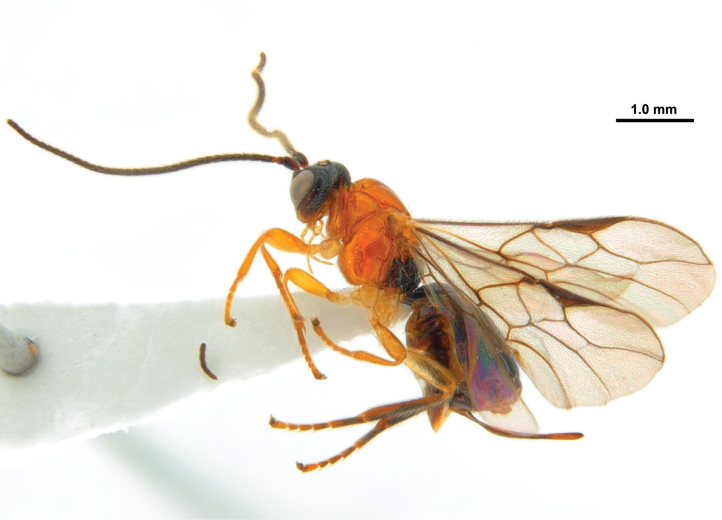
*Xynobiusbrevifemora* Han & van Achterberg, sp. nov., holotype, ♀, habitus, lateral.

**Figures 14–24.**
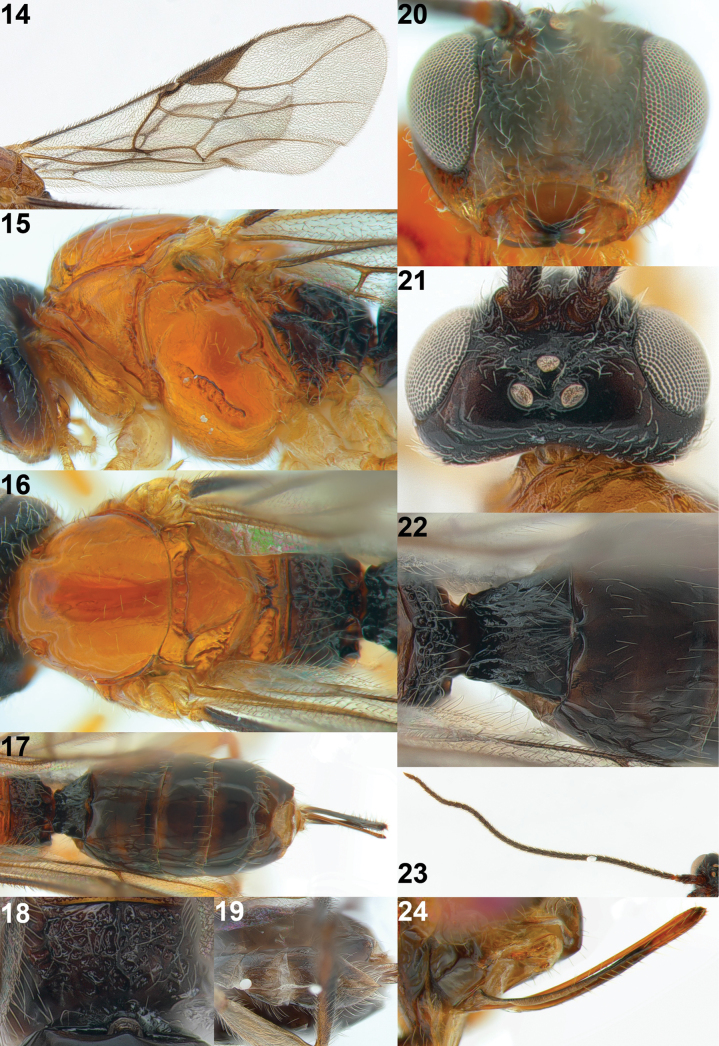
*Xynobiusbrevifemora* Han & van Achterberg, sp. nov., holotype, ♀ **14** wings **15** mesosoma, lateral view **16** mesosoma, dorsal view **17** metasoma, dorsal view **18** propodeum, dorsal view **19** hypopygium, ventral view **20** head, anterior view **21** head, dorsal view **22** 1^st^–3^rd^ metasomal tergites, dorsal view **23** antenna **24** ovipositor and its sheath, lateral view.

Apical third of antenna dark brown or black (Fig. 23); eye 2.4–2.7× longer than temple in dorsal view; middle lobe of mesoscutum largely glabrous and strongly shiny (Fig. 16); scutellum slightly convex; fore wing at most slightly infuscated (Fig. 14); pterostigma gradually narrowed apically, triangular; hind tarsus largely dark brown or brown (Fig. 28); first tergite approximately as long as wide apically (Fig. 26); second metasomal tergite smooth; setose part of ovipositor sheath 0.3× as long as fore wing, 2.2× first tergite and 1.4× hind tibia; all femora robust (Fig. 13); mesosoma except metapleuron and propodeum orange brown.

#### Description.

Female; length of body 4.0 mm, of fore wing 3.9 mm.

***Head*.** Antenna with 40 segments and 1.1× as long as body; third segment of antenna 1.9× longer than its width (Fig. 23); area between antennal sockets rugose; eye 2.7× longer than temple in dorsal view (Fig. 21); vertex and stemmaticum shiny, smooth and moderately setose; frons finely punctate and densely setose (Fig. 20); face punctate and densely short setose; clypeus 2.8× wider its maximum height (Fig. 20); ventral margin of clypeus slightly concave and sparsely setose; hypoclypeal depression present; length of maxillary palp nearly as long as height of head; malar sulcus absent; occipital carina absent dorsally; mandible triangular in lateral view, hardly twisted and gradually widened basally (Fig. 27).

***Mesosoma*.** Mesosoma 1.3× longer than its height; pronope absent (Fig. 21); pronotum with indistinctly crenulated groove posteriorly; mesopleuron largely shiny and smooth, but precoxal sulcus oblique and moderately crenulate; epicnemial area crenulate ventrally, remaining area smooth (Fig. 15); pronotal side largely smooth except crenulated groove anteriorly and posteriorly; mesopleural sulcus smooth; anterior groove of metapleuron crenulate; metapleuron coarsely punctate and sparsely setose posteriorly (Fig. 15); notauli absent on disc of mesoscutum but as a pair of short and deep impressions present anteriorly; mesoscutum shiny, smooth and with few setae, middle lobe largely glabrous (Fig. 16); medio-posterior depression of mesoscutum absent; scutellar sulcus narrow and crenulate (Fig. 16); scutellum largely shiny and smooth, rather flat in lateral view; propodeum sparsely setose with long medio-longitudinal carina connected to two longitudinal carinae posteriorly forming reversed Y posteriorly, no transverse carina and remaining area coarsely rugose (Figs 16–18).

***Wings*.** Fore wing (Fig. 14): pterostigma wide, triangular and narrowed apically ending before level of vein r-m; vein r nearly 0.6× longer than vein 2-SR; vein 1-SR+M sinuate; vein 3-SR sublinear with vein r, parallel with vein 2-M and 1.8× longer than vein 2-SR; vein 2-SR almost straight; vein SR1 curved upward, ~ 2.0× longer than vein 3-SR; vein 1-M curved; second submarginal cell narrow; r: 3-SR: SR1 = 5: 13: 25; vein m-cu distinctly antefurcal, converging to vein 1-M posteriorly; first subdiscal cell transverse; vein CU1b short. Hind wing (Fig. 14): vein m-cu absent; vein 1r-m 0.5× as long as vein 1-M; vein 2-M only pigmented.

***Legs*.** Hind femur 3.4× longer than its maximum width (Fig. 28).

**Figures 25–28.**
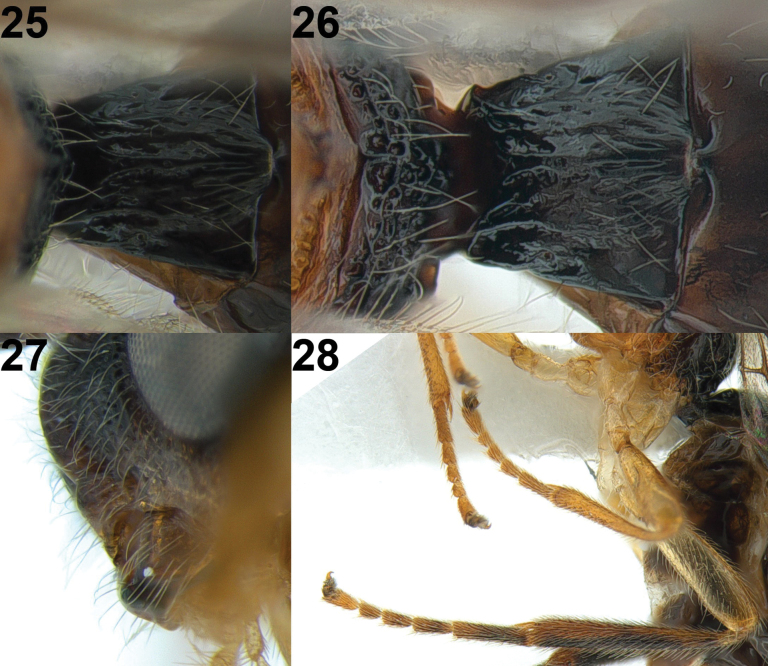
*Xynobiusbrevifemora* Han & van Achterberg, sp. nov., holotype, ♀ **25** 1^st^ metasomal tergite, dorsal view **26** 1^st^ metasomal tergite, dorso-posterior view **27** mandible, lateral view **28** hind leg, lateral view.

***Metasoma*.** Length of first metasomal tergite 1.1× its apical width; first tergite gradually widened apically and its surface with longitudinal striae medially, and remaining area shiny and smooth (Figs 22, 25, 26); dorsope present and surrounded by strongly curved dorsal carinae (Figs 22, 25, 26); second metasomal suture indistinctly indicated dorsally (Fig. 22); second tergite shiny and smooth except a pair of droplet-shaped impressions anteriorly; second tergite 0.6× as long as third tergite in dorsal view; following tergites shiny, smooth and moderately setose posteriorly (Fig. 17); hypopygium 0.4× as long as metasoma, rather acute apically and reaching apex of metasoma (Fig. 19); setose part of ovipositor sheath ~ 2.2× longer than first tergite and 0.3× as long as fore wing (Figs 13, 24).

***Colour*.** Body generally blackish to dark brown (Fig. 13); face and temple ventrally, mesoscutum, scutellum, metanotum, pronotum, mesopleuron, and ovipositor yellowish brown to orange (Figs 15, 16); palp, tegulae, legs (except hind tibia dorsally and tarsus) pale brown; narrowed band on fourth–sixth tergites anteriorly (Fig. 17) and apical segments of antenna brown; pterostigma and veins of wings dark brown.

#### Distribution.

South Korea.

#### Biology.

Unknown.

#### Etymology.

Named after the robust and comparatively short femora of the new species (Figs 13, 28); *brevis* is Latin for short.

#### Remarks.

The new species has a rather shallow dorsope bordered with strongly curved dorsal carinae basally, vein r much shorter than vein 2-SR and a large hypoclypeal depression; therefore, it belongs to the genus *Xynobius*. It has the reduced notauli (absent on mesoscutal disc and only distinctly impressed anteriorly), glabrous middle lobe of mesoscutum, a long medio-longitudinal carina on propodeum with two diverging longitudinal carinae posteriorly and remainder coarsely rugose, the short second submarginal cell of fore wing, median keel present between antennal sockets, second metasomal tergite relatively shorter than third tergite and relatively long setose part of ovipositor sheath. In the key by Tobias (1998), it runs to the subgenus Psyttalia Walker sensu Tobias by having two diverging medio-longitudinal carinae posteriorly on propodeum, short second metasomal tergite (0.7× as long as third metasomal tergite) and indistinctly indicated second metasomal suture. This new species is superficially similar to *P.spectabilis* van Achterberg, 2016, because they share the reduced medio-posterior depression of mesoscutum, vein r of fore wing sublinear with vein 3-SR, mesosoma yellowish brown to orange (except propodeum and metapleuron blackish to dark brown), pterostigma of fore wing distinctly triangular and reduced vein m-cu of hind wing. The new species has the dorsope present and dorsal carinae on first metasomal tergite not united (dorsope absent but dorsal carinae strong in its basal half and with depressed area below in *P.spectabilis*), ventral margin of clypeus strongly convex (slightly convex in *P.spectabilis*), median keel on frons between antennal sockets present (keel absent and frons behind antennal sockets rugose in *P.spectabilis*), with two longitudinal carinae on propodeum and partly coarsely rugose (smooth in *P.spectabilis*) and obtuse apex of hypopygium (acute apex in *P.spectabilis*). In the key by Fischer (1972), it runs to the subgenus Phlebosema Fischer, and to *Opiusfischeri* Papp, 1981. However, *O.fischeri* has no medio-longitudinal carinae on the propodeum (medio-longitudinal carina present in the new species), reduced median keel between antennal sockets (present), third segment of antenna more than 3.0× longer than its width (1.9× longer than its width), first metasomal tergite smooth (striate medially) and relatively short setose part of ovipositor sheath (~ 2.7× longer than first tergite).

Among the described Korean and Chinese species of *Xynobius*, the new species is similar to *X.gracilitergum* (Fischer, 1990) and *X.sulciferus* (Papp, 1967) because of sharing the slightly convex scutellum, length of eye 1.6–2.7× temple in dorsal view, vein m-cu of fore wing antefurcal (but slightly so in *X.sulciferus*), wing membrane at most slightly infuscated and second metasomal tergite smooth. *Xynobiusbrevifemora* differs from both by having no medio-posterior depression of mesoscutum (present in both species), first tergite approximately as long as its apical width (1.7–2.2× in both species), setose part of ovipositor sheath ~ 1.4× longer than hind tibia (shorter than length of hind tibia in both species), femora robust (femora more slender in both species) and mesosoma (except metapleuron and propodeum) orange-brown (black in both species).

### ﻿ Xynobius duoferus

Taxon classificationAnimaliaHymenopteraBraconidae

Han & van Achterberg sp. nov.

B6013E51-9C3C-559D-B467-F2A8DDA955B8

https://zoobank.org/7F8C078F-94F4-4D71-BB49-3DB46F8C947E

Figs 29 , 30–41

#### Type material.

***Holotype*.** ♀ (NIBR), “South Korea: Jangam Cave, Pyeongchang-gun, Gangwon prov., 37°23'54.2"N, 128°25'24.2"E, 11.vii.2020, SW [= collected by sweeping], Hyojoong Kim leg., KSNU”.

**Figure 29.**
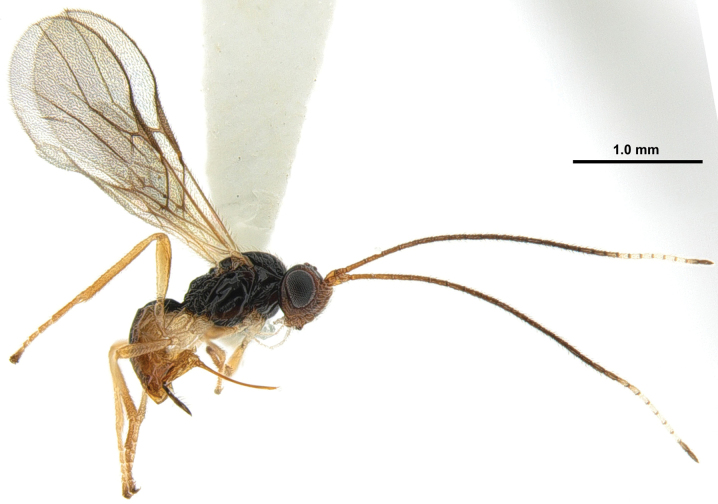
*Xynobiusduoferus* Han & van Achterberg, sp. nov., holotype, ♀, habitus, lateral.

#### Diagnosis.

Dorsope distinct (Fig. 37); first metasomal tergite with straight longitudinal striae; notauli complete and narrowly crenulate (Fig. 32); mesoscutum largely smooth and sparsely setose medially; second tergite striate-rugose medially; 20^th^ to 26^th^ antennal segments of ♀ white followed by two dark apical segments.

**Figures 30–41.**
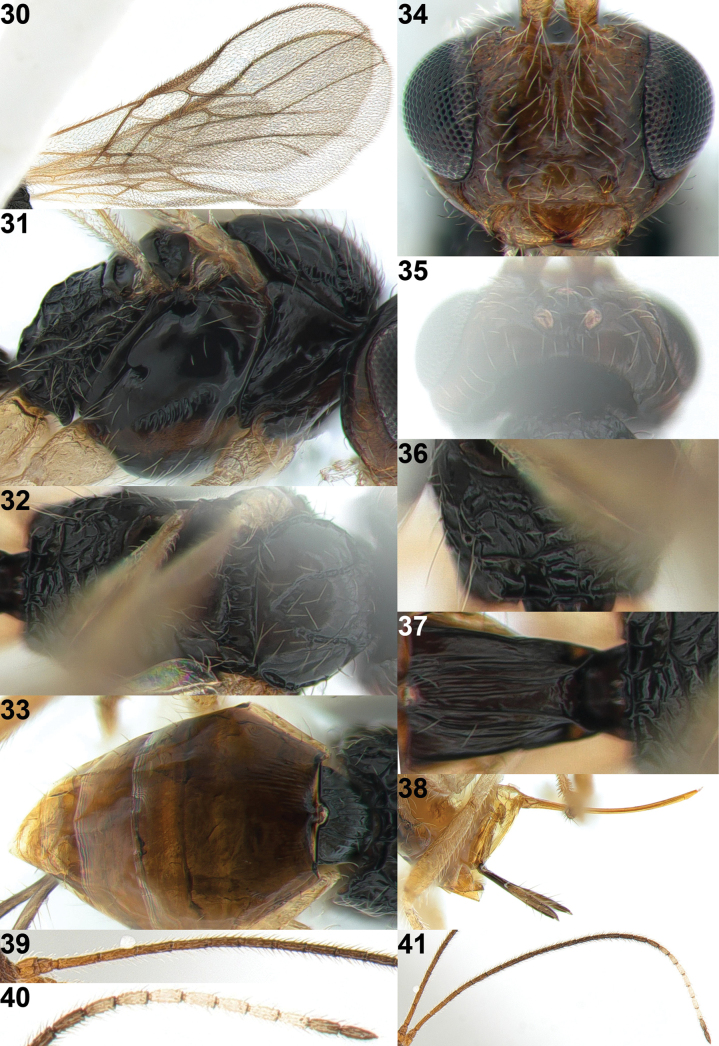
*Xynobiusduoferus* Han & van Achterberg, sp. nov., holotype, ♀ **30** wings **31** mesosoma, lateral view **32** mesosoma, dorsal view **33** metasoma, dorsal view **34** head, anterior view **35** head, dorsal view **36** propodeum, dorsal view **37** 1^st^ metasomal tergite, dorsal view **38** ovipositor and its sheath, lateral view **39** basal part of antenna **40** apical part of antenna **41** antenna.

#### Description.

Female; length of body 2.0 mm, of fore wing 2.5 mm.

***Head*.** Antenna with 28 segments and 1.6× longer than body; third segment of antenna 5.8× longer than wide and 1.1× longer than fourth segment (Figs 39, 41); depression of frons present near antennal sockets (Fig. 34); eye ~ 2.5× longer than temple in dorsal view (Fig. 35); frons and vertex smooth, glabrous and moderately setose; face largely shiny, smooth and densely setose, but granulate latero-dorsally; median keel present; clypeus twice wider than its maximum height (Fig. 34); clypeus semi-circular, moderately setose, and ventral margin of clypeus straight and above upper level of condyles of mandible; hypoclypeal depression present; length of maxillary palp nearly 0.9× as long as height of head; malar sulcus absent; occipital carina absent dorsally; mandible triangular in lateral view, hardly twisted and gradually widened basally (Fig. 34).

***Mesosoma*.** Mesosoma 1.4× longer than its height; pronotal side largely smooth and smooth groove present along its ventral margin; propleuron smooth and sparsely setose, without transverse carinae; mesopleuron largely smooth and sparsely setose antero-dorsally and postero-ventrally, but precoxal sulcus oblique, medium-sized and densely crenulate; epicnemial area smooth (Fig. 31); mesopleural sulcus smooth; anterior groove of metapleuron smooth; metapleuron reticulate-rugose and moderately setose (Fig. 31); notauli complete on disc of mesoscutum and narrowly crenulate; mesoscutum smooth, largely glabrous but middle lobe sparsely setose (Fig. 32); medio-posterior depression of mesoscutum round and shallow; scutellar sulcus wide and crenulate (Fig. 32); scutellum largely smooth and glabrous, rather flat in lateral view and protruding above level of mesoscutum; propodeum rugose with long medio-longitudinal carina, transverse carinae, and areola, remainder of propodeum largely smooth (Figs 32, 36).

***Wings*.** Fore wing (Fig. 30): pterostigma triangular and rather directly narrowed apically; vein 1-SR+M almost straight; vein 3-SR sublinear with vein r, converging with vein 2-M and 1.6× longer than vein 2-SR; vein 2-SR almost straight; vein SR1 straight, 2.0× longer than vein 3-SR; vein 1-M straight; r: 3-SR: SR1 = 5: 40: 84; vein m-cu distinctly postfurcal, converging to vein 1-M posteriorly and angled with vein 2-M; first subdiscal cell transverse and closed; vein CU1b present. Hind wing (Fig. 30): vein m-cu absent; vein 1r-m 0.8× as long as vein 1-M; vein 2-M pigmented.

***Legs*.** Hind femur 4.5× longer than its maximum width (Fig. 29).

***Metasoma*.** First metasomal tergite 1.5× longer than its apical width; first tergite gradually widened apically and its surface densely longitudinally striate postero-medially, and remainder of tergite shiny and smooth (Fig. 37); dorsope present and surrounded by strongly curved dorsal carinae (Fig. 37); second metasomal suture absent dorsally (Fig. 33); second tergite striate-rugose medially except a pair of droplet-shaped impressions anteriorly; following tergites shiny, smooth and moderately setose posteriorly (Fig. 33); setose part of ovipositor sheath ~ 1.2× longer than first tergite and 0.1× as long as fore wing (Fig. 38).

***Colour*.** Body generally brown to black (Fig. 29); entire head, transverse band below the precoxal sulcus, tarsi, inside of dorsope, and first and second tergites dorsally dark brown; legs, remaining tergites, ovipositor, and basal segments (1^st^–6^th^) of antenna brown; palp pale yellowish or white; apical segments (20^th^–26^th^) of antenna white to white-brown; pterostigma and veins of wings pale brown; wings subhyaline.

#### Distribution.

South Korea.

#### Biology.

Unknown.

#### Etymology.

Name derived from *duo* (Latin for two) and -*fero* (suffix in Latin meaning carrying or having), because of the two apical dark antennal segments.

#### Remarks.

This species runs to *Xynobiusnotauliferus* Li & van Achterberg, 2013 in the key by Li et al. (2013). It differs by having the length of maxillary palp 0.9× height of head (1.4× in *X.notauliferus*), face smooth, but granulate latero-dorsally (smooth), clypeus twice wider than high (1.6× wider than its maximum height), first metasomal tergite 1.5× longer than its apical width (length 1.3×), antenna of ♀ with two apical antennal segments dark brown (6 or 7 such segments), pterostigma directly narrowed distally (gradually narrowed) and outer side of hind femur without brownish patch (with patch).

### ﻿ Xynobius stipitoides

Taxon classificationAnimaliaHymenopteraBraconidae

Han & van Achterberg sp. nov.

FFA119C3-D72F-5072-9E92-F3C5FC87BD11

https://zoobank.org/D7935E14-BA59-4F89-93F6-419D42EAB220

Figs 42 , 43–53

#### Type material.

***Holotype*.** ♀ (KSNU), “South Korea: Forahn house, Ongpo-ri, Hallim, Jeju, Jeju Island, 33°12'51.1"N, 126°15'04.0"E, 16.v.2019, SW [= collected by sweeping], Hyojoong Kim leg., KSNU”.

**Figure 42.**
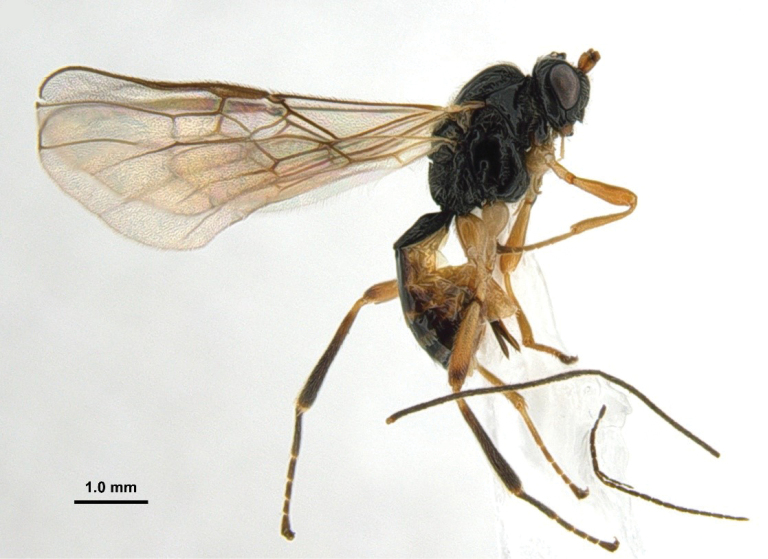
*Xynobiusstipitoides* Han & van Achterberg, sp. nov., holotype, ♀, habitus, lateral.

#### Diagnosis.

**Figures 43–53.**
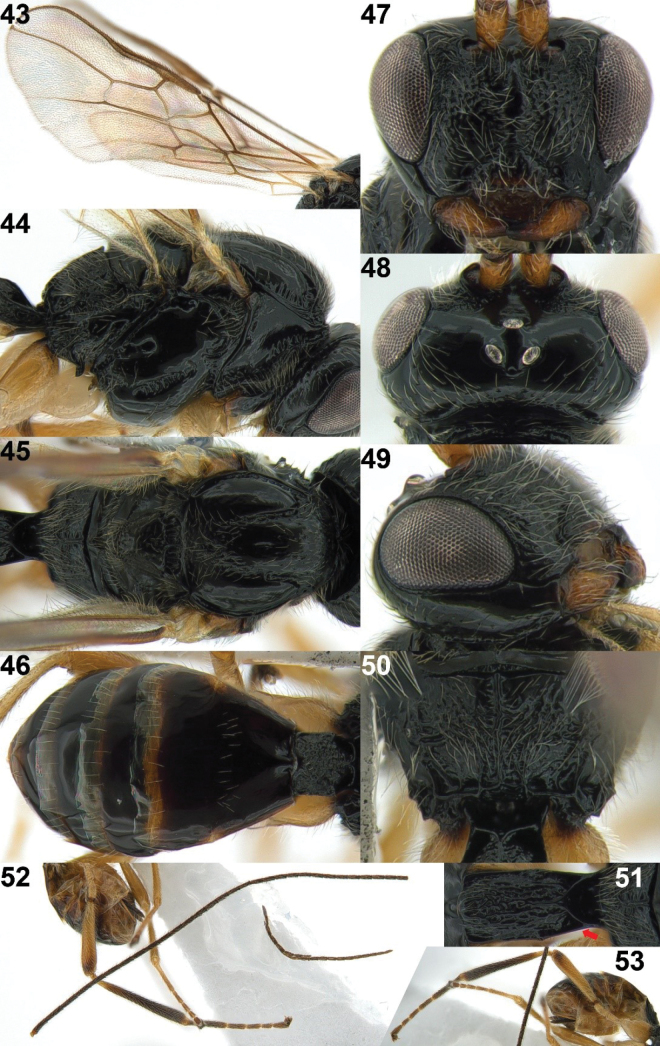
*Xynobiusstipitoides* Han & van Achterberg, sp. nov., holotype, ♀ **43** wings **44** mesosoma, lateral view **45** mesosoma, dorsal view **46** metasoma, dorsal view **47** head, anterior view **48** head, dorsal view **49** head, lateral view **50** propodeum, dorsal view **51** 1^st^ metasomal tergite, dorsal view **52** antenna **53** hind leg and ovipositor, lateral view. The red arrow points to the dorsope.

Pterostigma slightly widened apically (Fig. 43); maxillary palp 1.5× longer than height of head; notauli present up to middle of mesoscutum and narrowly crenulate, mesoscutum medio-posteriorly and scutellum punctate (Fig. 45); precoxal sulcus oblique and moderately crenulate medially (Fig. 44); vein SR1 of fore wing 2.7× as long as vein 3-SR; first tergite subparallel-sided and nearly twice longer than its apical width (Fig. 51); second tergite shiny and smooth (Fig. 46); setose part of ovipositor sheath slightly shorter than first tergite (Fig. 52); hind tibia (except ivory base) and tarsus dark brown.

#### Description.

Female; length of body 5.9 mm, of fore wing 4.6 mm.

***Head*.** Antenna with 48 segments and 1.2× as long as body; third segment 3.5× longer than its width and 0.9× as long as fourth segment (Fig. 52); eye 1.7× longer than temple in dorsal view (Fig. 48); temple smooth and moderately setose; vertex, stemmaticum and frons shiny and smooth; face densely punctate and setose but granulate dorso-laterally; median keel present on face (Fig. 47); width of clypeus 1.9× its maximum height; clypeus punctate and protruding anteriorly in lateral view (Figs 42, 49); ventral margin of clypeus above upper level of condyli of mandibles and densely setose; hypoclypeal depression rather deep (Fig. 47); maxillary palp 1.5× longer than height of head; malar sulcus rather deep and curved anteriorly in lateral view (Figs 42, 49); occipital carina protruding dorsally in lateral view; interrupted dorso-medially (Fig. 48); mandible twisted and triangular in lateral view and gradually widened basally (Fig. 49).

***Mesosoma*.** Mesosoma 1.4× longer than its height (Fig. 44); pronope absent (Figs 45, 48); propleuron flange largely smooth and protruding posteriorly with oblique carina (Fig. 44); mesopleuron largely shiny and smooth, but precoxal sulcus oblique and moderately crenulate medially; epicnemial area crenulate ventrally, remaining area smooth; pronotal side largely smooth except crenulated groove anteriorly and ventrally; mesopleural sulcus crenulate and narrowed dorsally; anterior groove of metapleuron narrow and crenulate; metapleuron flange distinctly protruding ventrally (Fig. 44); metapleuron reticulate-rugose and moderately setose posteriorly and remainder of metapleuron smooth; notauli present anteriorly up to middle of mesoscutum and narrowly crenulate, medio-posteriorly mesoscutum densely punctate and with setae (Fig. 45); medio-posterior depression of mesoscutum sublinear, shallow and surroundings distinctly punctate; scutellar sulcus wide, distinctly and densely crenulate; scutellum sparsely punctate and setose medially, posteriorly densely punctate and rather flat in lateral view; propodeum shiny and densely setose medially with long medio-longitudinal carina and indistinctly transverse carina medially (together cross-shaped) and remaining area mainly coarsely rugose (Figs 45, 50).

***Wings*.** Fore wing (Fig. 43): pterostigma narrow, elongated, sublinear and slightly widened apically, ending after level of vein r-m (Fig. 43); vein r 0.4× longer than vein 2-SR; vein 1-SR+M sinuate; vein 3-SR angled with vein r, parallel with vein 2-M and 1.5× longer than vein 2-SR; vein 2-SR slightly curved upward; vein SR1 curved upward, 2.5× longer than vein 3-SR; vein 1-M straight; first subdiscal cell rather transverse; r: 3-SR: SR1 = 5: 16: 42; vein m-cu distinctly postfurcal; vein CU1b completely present. Hind wing: vein m-cu absent; vein 1r-m 0.6× as long as vein 1-M; vein 2-M incompletely pigmented.

***Legs*.** Hind femur 4.7× longer than its maximum width (Fig. 53); hind leg long and densely setose.

***Metasoma*.** Length of first metasomal tergite ~ 2.0× its apical width (Fig. 51); first tergite slightly widened medially and parallel-sided posteriorly, setose, dorsal carinae converging to short medio-longitudinal carina at basal third and remaining area reticulate-rugose; dorsope distinct (Figs 45, 46, 50, 51); second metasomal suture absent dorsally (Fig. 46); second tergite shiny and smooth with a pair of impressions anteriorly; following tergites shiny, smooth and moderately setose posteriorly; setose part of ovipositor sheath 0.8× and 0.1× as long as first tergite and fore wing, respectively (Figs 42, 52, 43).

***Colour*.** Body generally black; antenna, ovipositor sheath, and tibia, tarsus, and femur of hind leg dark brown (Figs 52, 53); antennal sockets, ventral margin of clypeus, mandibles, and legs (Figs 52, 53; except hind tibia and tarsus) brownish yellow; palpi and tegulae pale yellowish; ovipositor, narrowed band on third–sixth tergites posteriorly and spot of second tergite latero-posteriorly, yellowish brown (Fig. 46); pterostigma and veins of wings greyish brown.

#### Distribution.

South Korea

#### Biology.

Unknown.

#### Etymology.

Name is a combination of the specific name *stipitatus* and *oides* (Latin for resembling) because the new species is similar to *Opiusstipitatus* Tobias.

#### Remarks.

This species has a distinct dorsope, ventral margin of clypeus above upper level of mandibular condyles and a large hypoclypeal depression; therefore, it belongs to the genus *Xynobius*. It has a curved malar suture in lateral view, reduced notauli (narrowly crenulated anteriorly and absent on posterior half of mesoscutal disc), largely shiny and smooth mesoscutum except some punctures anteriorly and around medio-posterior depression, elliptical depression medio-posteriorly on middle lobe of mesoscutum, a long medio-longitudinal carina with indistinct transverse carina on propodeum, and hind leg with long, evenly and conspicuous setae. In the key by Tobias (1998), it runs to Opius (Xynobius) stipitatus Tobias, 1998 (Figs 65–75), by having the scutellum sculptured, the mesoscutum largely smooth and the pterostigma more or less widened apically. However, it differs by having the narrowly crenulated notauli up to middle of mesoscutum (notauli absent on mesoscutal disc in O. (X.) stipitatus, except for shallow depressions at imaginary notaulic courses), middle lobe of mesoscutum shallowly punctate and densely setose medio-posteriorly (smooth and glabrous), precoxal sulcus crenulated (precoxal sulcus smooth), groove on pronotal side narrowly crenulated and without setae ventrally (crenulated groove rather wide and densely setose ventrally), propodeum with long medio-longitudinal carina and indistinct transverse carina medially (with short medio-longitudinal carina, coarse transverse carinae and indistinct areola), length of first metasomal tergite ~ 2.0× its apical width (1.3× longer than its apical width), dorsal carinae forming a short medio-longitudinal carina on first tergite (longitudinal carinae remain separated), and third–sixth tergites with distinct brown band posteriorly (only third tergite with obsolescent brown band posteriorly). Among the Chinese species it shares the shape of the pterostigma, the setose and punctate medio-posterior area of the mesoscutum, the antenna of ♀ with ~ 48 segments, and the largely punctate face (except for the smooth medial ridge) with *Xynobiusrugosulcus* (Wu & Chen, 2005), comb. nov. (it was described as *Eurytenesrugosulcus* but it lacks the typical derived venation of *Eurytenes* s. str.). The new species differs by having the posterior half of the notauli reduced (notauli nearly complete in *Xynobiusrugosulcus*), middle lobe of mesoscutum shallowly punctate and densely setose medio-posteriorly (with a pair of grooves parallel to notauli), scutellum punctate medially (scutellum smooth medially), face coarsely and more densely punctate submedially (finer and sparsely punctate submedially), precoxal sulcus distinctly crenulated (precoxal sulcus slightly punctate), propodeum with long medio-longitudinal carina and indistinct transverse carina medially (with coarse transverse carinae and areola, and without medio-longitudinal carina), vein m-cu of hind wing absent (vein m-cu of hind wing as an unpigmented fold), and length of first metasomal tergite 1.9× its apical width (1.6× longer than its apical width).

### ﻿ Xynobius geniculatus

Taxon classificationAnimaliaHymenopteraBraconidae

(Thomson, 1895)

A6DB0065-CC7F-5688-8CB3-1B5ACFEE15E7

Figs 54 , 55–64

Opius geniculatus Thomson, 1895: 2179.
Opius (Nosopoea) geniculatus : Fischer 1972: 282–284; Papp 1981b: 44–59.
Opius (Allotypus) geniculatus : Tobias and Jakimavičius 1986: 63.
Opius albicoxis Marshall, 1898: 236; Fischer 1967: 143 (as synonym of O.geniculatus Thomson), 1972: 282.

#### Material examined.

1 ♀ (KNA), “South Korea: DMZ Botanical Garden, Mandae-ri, Haean, Yanggu, Gangwon prov., 38°15'09.3"N, 128°06'40.6"E, 17.x.2017–17.xi.2017, MT [= Malaise trap]”.

#### Diagnosis.

**Figure 54.**
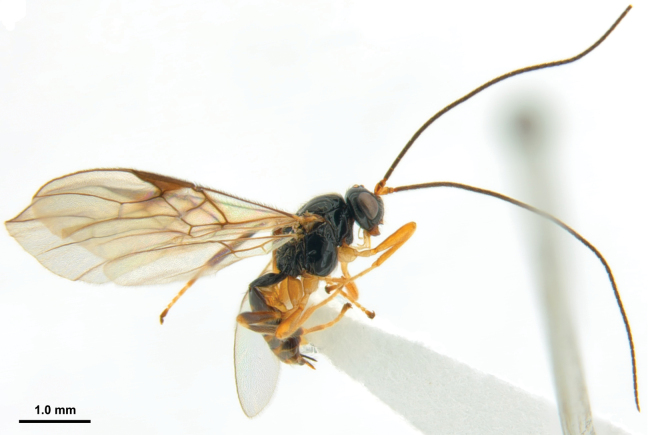
*Xynobiusgeniculatus* (Thomson), ♀, habitus, lateral.

**Figures 55–64.**
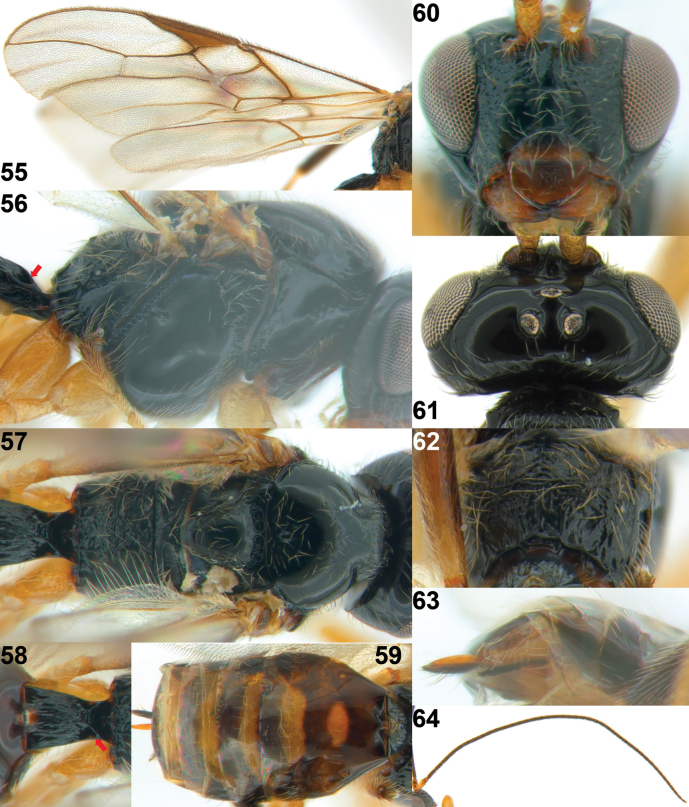
*Xynobiusgeniculatus* (Thomson), ♀, South Korea **55** wings **56** mesosoma, lateral view **57** mesosoma, dorsal view **58** 1^st^ metasomal tergite, dorsal view **59** metasoma, dorsal view **60** head, anterior view **61** head and pronotum, dorsal view **62** propodeum, dorsal view **63** ovipositor and sheath, latero-ventral view **64** antenna. The red arrow indicates the dorsope.

Antennal segments of ♀ 38–44; area below pterostigma with brownish patch (Figs 54, 55), rarely obsolescent; precoxal sulcus smooth; mesoscutum largely glabrous, but middle lobe sparsely setose, notauli absent on disc, only anteriorly deeply impressed and medio-posterior depression distinct; pterostigma triangular; vein M+CU1 of fore wing largely sclerotised (Fig. 55); second metasomal tergite bicoloured (dark brown and with a pale yellowish patch medially); hind tarsus (except telotarsus) pale yellowish or ivory; second submarginal cell of fore wing long (Fig. 55); area around medio-posterior depression of mesoscutum finely punctate or punctulate; at least apex of hind femur dark brown; no pronope; setose part of ovipositor sheath 0.6× as long as first metasomal tergite.

#### Re-description.

Female; length of body 3.6 mm, of fore wing 4.1 mm.

***Head*.** Antenna with 44 segments and 1.5× as long as body (Fig. 64); third segment of antenna 2.6× longer than wide, ~ 1.1× longer than fourth segment of antenna; eye 2× longer than temple in dorsal view (Fig. 61); stemmaticum shiny and smooth; vertex shiny, smooth and moderately setose posteriorly; frons with depression medially and remainder shiny and smooth; face densely punctate and setose, median keel present up to between antennal sockets (Fig. 60); clypeus 2.4× wider than its maximum height; clypeus punctate and moderately setose, rather flat in lateral view; hypoclypeal depression present; malar sulcus straight; occipital carina absent medio-dorsally; mandible slightly curved apically, triangular in lateral view and gradually widened basally.

***Mesosoma*.** Mesosoma 1.5× longer than its height (Fig. 56); pronope absent (Figs 57, 61); propleuron largely smooth and propleuron flange present posteriorly (Fig. 56); mesopleuron largely shiny and smooth, including narrow precoxal sulcus; epicnemial area distinctly crenulate; pronotal side largely smooth with crenulated groove anteriorly and posteriorly; mesopleural sulcus crenulate; anterior groove of metapleuron crenulate; metapleuron largely shiny, smooth and moderately setose along grooves; notauli absent on disc of mesoscutum (Fig. 57); mesoscutum shiny, smooth and densely setose medially; scutellum slightly punctate and setose; medio-posterior depression of mesoscutum round; scutellar sulcus distinctly crenulate, medium-sized and curved; propodeum moderately setose with short medio-longitudinal carina anteriorly, indistinct transverse carina and areola, remainder of propodeum shiny and largely rugose (Figs 57, 62).

***Wings*.** Fore wing (Fig. 55): pterostigma triangular with dark spot below pterostigma; vein 1-SR+M sinuate; vein 3-SR angled with vein r, parallel with vein 2-M and ~ 2× longer than vein 2-SR; vein 2-SR straight and oblique; vein SR1 curved upward, nearly 1.8× longer than vein 3-SR; r: 3-SR: SR1 = 5: 46: 84; vein m-cu postfurcal; second submarginal cell elongated; vein CU1b medium-sized. Hind wing: vein m-cu pigmented and curved basally; vein 1r-m 0.6× as long as vein 1-M; vein 2-M pigmented.

***Legs*.** Hind femur 4.6× longer than its maximum width (Fig. 54).

***Metasoma*.** First metasomal tergite 1.3× longer than its apical width (Fig. 58); first tergite slightly widened apically and its surface with longitudinal striae medially and remaining area shiny and smooth; dorsope distinct (Figs 57, 58); second metasomal suture indistinctly indicated (Fig. 59); second tergite shiny and smooth, with a pair of oblique depressions anteriorly; second and following tergites shiny, smooth, with transverse band of setae posteriorly; setose part of ovipositor sheath 0.6× as long as first metasomal tergite and nearly 0.07× as long as fore wing (Fig. 63).

***Colour*.** Body black (Fig. 54); clypeus ventrally, mandible, and ovipositor brown; flagellar segments of antenna, femur, and tibia of hind leg and tarsal claw dark brown; scape of antenna, pterostigma, vein of wings and spot below pterostigma, tegulae, and remainder of legs brown; palpi pale yellowish; posterior band of third–sixth metasomal tergites brown or yellowish brown.

#### Distribution.

South Korea (new record), Europe, Eastern/Western Palearctic region.

#### Biology.

Parasitoid of *Trypetaimmaculata* (Macquart, 1835) and *Stemonoceracornuta* (Scopoli, 1771) (Tephritidae) (Yu et al. 2016).

#### Remarks.

This species runs in the key by Tobias (1998) to *Opiusgeniculatus* Thomson, because of having the distinct medio-posterior depression of mesoscutum, smooth precoxal sulcus, distinct hypoclypeal depression, vein m-cu of fore wing weakly postfurcal, hind femur 4.6× longer than its width, antenna with 44 segments, pterostigma short and triangular, mesoscutum rather densely setose, vein 3-SR of fore wing twice longer than vein 2-SR, a brownish spot below pterostigma and brownish band posteriorly at third–sixth metasomal tergites.

**Figures 65–75.**
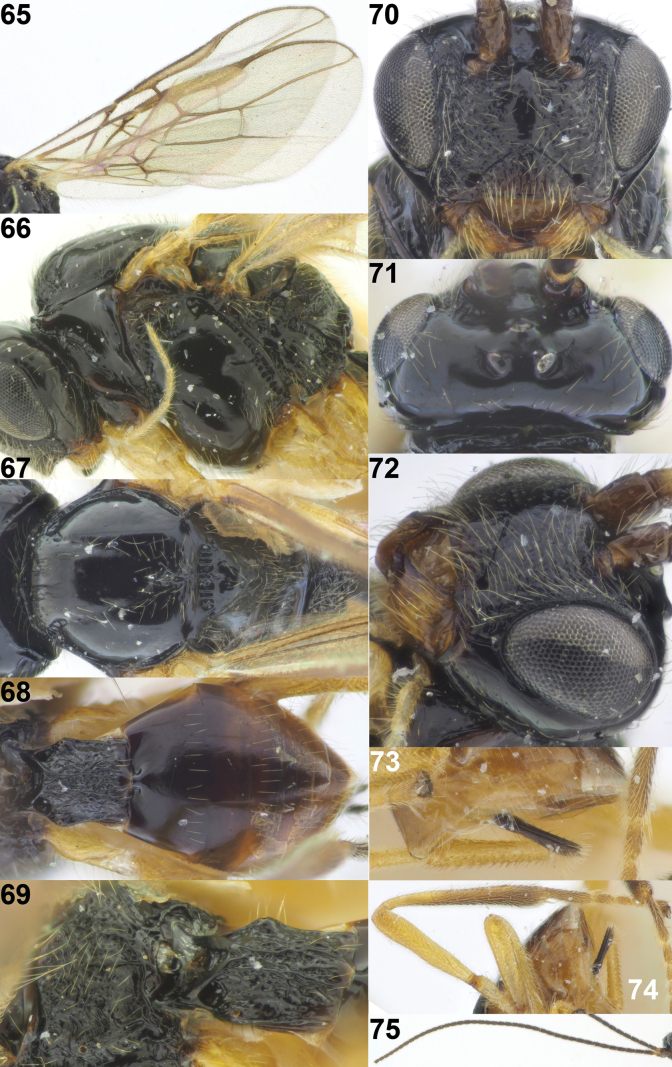
*Xynobiusstipitatus* (Tobias), holotype, ♀, Russia **65** wings **66** mesosoma lateral **67** mesosoma dorsal **68** metasoma dorsal **69** propodeum and 1^st^ metasomal tergite latero-dorsal **70** head anterior **71** head and pronotum dorsal **72** head latero-anterior **73** ovipositor sheath lateral **74** hind leg **75** antenna. Photographs: Konstantin Samartsev.

## Supplementary Material

XML Treatment for Xynobius

XML Treatment for Xynobius azonius

XML Treatment for Xynobius brevifemora

XML Treatment for Xynobius duoferus

XML Treatment for Xynobius stipitoides

XML Treatment for Xynobius geniculatus

## ﻿Additional information

### Author ORCIDs

Yunjong Han https://orcid.org/0000-0003-2757-7785

Cornelis van Achterberg https://orcid.org/0000-0002-6495-4853

Hyojoong Kim https://orcid.org/0000-0002-1706-2991

### Data availability

All of the data that support the findings of this study are available in the main text.
